# Histopathological biomarkers of immunotherapy outcome in advanced colorectal cancer: a multicentre retrospective study

**DOI:** 10.3389/fonc.2026.1811658

**Published:** 2026-07-06

**Authors:** Jianying Chen, Yanzhi Chen, Zhigang Chen

**Affiliations:** 1 Department of Histology and Embryology, Jiangxi Medical College, Shangrao, China; 2 Department of Pathology and Pathophysiology, Jiangxi Medical College, Shangrao, China; 3 Department of Oncology, Shangrao People’s Hospital, Shangrao, China

**Keywords:** colorectal neoplasms, immunotherapy, prognosis., tumor-infiltrating lymphocytes, tumor-stroma ratio

## Abstract

**Background:**

Immune checkpoint inhibitors (ICIs) have transformed the treatment of advanced colorectal cancer (aCRC), but clinical benefit remains largely confined to patients with deficient mismatch repair (dMMR). Responses among patients with proficient mismatch repair (pMMR) are heterogeneous, underscoring the need for accessible biomarkers that can refine patient stratification beyond MMR status. Histopathological features on routine hematoxylin and eosin (H&E)-stained slides may reflect the immune and stromal architecture of the tumor microenvironment. This study investigated the prognostic value and treatment-outcome associations of standardized stromal tumor-infiltrating lymphocytes (sTILs), tumor-stroma ratio (TSR), and tumor budding (TB) in patients with aCRC treated with ICIs.

**Methods:**

This retrospective multicentre study included 210 patients with pathologically confirmed aCRC who received PD-1/PD-L1–based immunotherapy between January 2021 and June 2025. H&E-stained sections were independently assessed by two blinded pathologists. The primary endpoint was progression-free survival (PFS), with objective response rate (ORR) and overall survival (OS) as secondary endpoints. Survival outcomes were analyzed using Kaplan–Meier estimates and Cox proportional hazards models. Inter-observer agreement was evaluated using Cohen’s kappa.

**Results:**

At a median follow-up of 22.4 months, high sTILs (≥20%) were associated with a significantly higher ORR than low sTILs (47.2% vs. 15.2%, P<0.001). Patients with low stromal content (TSR ≥50%) experienced longer median PFS compared with those with high stromal content (10.8 vs. 5.2 months; HR 0.48, 95% CI 0.35–0.66; P<0.001). Within the pMMR subgroup (n=162), the combination of high sTILs and low TSR identified an immune-active phenotype with an ORR of 38.5% versus 6.1% in patients with neither feature. Multivariable analysis confirmed high sTILs (HR 0.47, 95% CI 0.26–0.84; P = 0.011) and high-grade tumor budding (HR 2.03, 95% CI 1.39–2.96; P<0.001) as independent prognostic factors for PFS, while TSR demonstrated prognostic value in univariate analysis only.

**Conclusions:**

Standardized assessment of routine H&E-stained histopathological features provides clinically relevant prognostic and outcome-stratifying information in ICI-treated patients in aCRC. These cost-effective biomarkers may complement molecular testing, particularly for stratifying pMMR patients in real-world immunotherapy settings.

## Introduction

Colorectal cancer remains one of the leading causes of cancer-related mortality worldwide, and a substantial proportion of patients present with advanced or metastatic disease at diagnosis (1, 2). The advent of immune checkpoint inhibitors (ICIs) targeting the programmed cell death 1 (PD-1)/programmed death-ligand 1 (PD-L1) axis has profoundly altered the therapeutic landscape of advanced colorectal cancer (aCRC), but the clinical benefit is largely restricted to tumors with deficient mismatch repair (dMMR) or high microsatellite instability (MSI-H) (3–5). In contrast, the majority of aCRC patients harbor proficient mismatch repair (pMMR) tumors, which are generally resistant to ICIs, resulting in heterogeneous and often disappointing clinical outcomes (6, 7). This marked disparity underscores an urgent need for reliable, accessible biomarkers capable of refining patient selection beyond MMR status alone.

Current biomarker strategies for predicting immunotherapy response in colorectal cancer predominantly rely on molecular profiling, including tumor mutational burden, gene expression signatures, and multiplex immune phenotyping (8–10). While biologically informative, these approaches are costly, technically demanding, and not routinely available in many clinical settings. Moreover, they may fail to capture the spatial organization of the tumor microenvironment (TME), which plays a critical role in shaping anti-tumor immune responses (11, 12). Routine hematoxylin and eosin (H&E)–stained slides, by contrast, are universally available and preserve the architectural context of tumor–immune interactions, offering a potential yet underutilized source of prognostic and outcome-stratifying information.

Accumulating evidence suggests that histopathological features observable on H&E slides—such as stromal tumor-infiltrating lymphocytes (sTILs), tumor–stroma ratio (TSR), and tumor budding—reflect key biological processes including immune activation, stromal remodeling, and epithelial–mesenchymal transition (13–16). These parameters have demonstrated prognostic value in colorectal cancer and other solid tumors, but their role as outcome-stratifying biomarkers in immunotherapy-treated populations remains insufficiently explored, particularly in real-world pMMR cohorts (17, 18). While the prognostic value of TILs has been extensively described in surgically treated colorectal cancer, their integration with TSR and tumor budding as practical H&E-based outcome-stratifying markers in advanced ICI-treated colorectal cancer, particularly pMMR disease, remains insufficiently defined. We therefore conducted a multicentre retrospective study to systematically evaluate whether standardized histopathological features could stratify treatment outcomes and survival in patients with aCRC receiving ICIs, with a specific focus on identifying immune-responsive subgroups within pMMR disease.

## Methods

### Study design and patient selection

This retrospective multicentre cohort study was conducted across three tertiary hospitals and was approved by the Ethics Committee of Shangrao People’s Hospital (Ethics Approval No. 2025-Medical Ethics-073). A total of 245 patients with advanced colorectal cancer (aCRC) who received immune checkpoint inhibitors (ICIs) between January 2021 and June 2025 were screened. After applying predefined inclusion and exclusion criteria, 210 patients were included in the final analysis. As this was a retrospective study, the sample size reflects the total eligible population within the defined enrolment period rather than a prospectively calculated target. A *post hoc* power analysis confirmed that n=210 with the observed PFS event rate (approximately 74%) and primary effect size (HR 0.47 for high vs. low sTILs) provides approximately 83% power (two-sided α=0.05) in a multivariable Cox model adjusted for six covariates, meeting established standards for survival analyses of this type.

The inclusion criteria were as follows: (1) histologically confirmed colorectal adenocarcinoma; (2) metastatic or unresectable disease; (3) receipt of at least two cycles of PD-1/PD-L1 inhibitor therapy (e.g., Sintilimab, Tislelizumab, or Camrelizumab), administered as monotherapy or in combination with other agents; and (4) availability of high-quality hematoxylin and eosin (H&E)–stained slides obtained from either the primary tumor or metastatic lesions.

Patients were excluded if they met any of the following criteria: (1) presence of other concurrent malignancies; (2) history of autoimmune disease requiring systemic immunosuppressive therapy; or (3) insufficient pathological material for standardized histopathological assessment.

### Clinical data collection

Clinical and demographic variables, including age, sex, primary tumor location, TNM stage (according to the AJCC 8th edition), and mismatch repair (MMR) status, were extracted from electronic medical records. MMR status was determined by immunohistochemistry (IHC) assessment of MLH1, MSH2, MSH6, and PMS2 expression.

Treatment response was evaluated by independent radiologists at intervals of 8–12 weeks using the Response Evaluation Criteria in Solid Tumors (RECIST), version 1.1. Treatment regimens were classified as ICI monotherapy or ICI-based combination therapy (with chemotherapy, anti-angiogenic agents, or dual ICI combinations as partner agents). Line of therapy (first-line, second-line, or third-line and beyond) was recorded for each patient and included as a covariate in sensitivity analyses of the multivariable Cox model to account for potential confounding from treatment heterogeneity.

### Histopathological assessment

All H&E-stained tissue sections (4 μm thickness) were independently reviewed by two senior gastrointestinal pathologists who were blinded to clinical outcomes and molecular characteristics.

### Stromal tumor-infiltrating lymphocytes

Stromal tumor-infiltrating lymphocytes (sTILs) were assessed as a continuous variable in accordance with the standardised methodology recommended by the International TILs Working Group (28, 29). Scoring was performed within the borders of the invasive tumor, with evaluation restricted to the stromal compartment. Areas of necrosis, hemorrhage, and crush artifacts were excluded from analysis.

High sTILs were defined as ≥20%, based on optimal threshold identification using X-tile software applied to the present cohort (optimized for PFS, AUC = 0.71 by time-dependent ROC analysis at 12 months), a threshold that is also consistent with commonly applied cut-off values reported in prior studies of sTILs in gastrointestinal malignancies. To assess the robustness of this threshold, sensitivity analyses were performed using alternative cut-offs of ≥10% and ≥30%, as well as a continuous-variable Cox model in which sTILs were entered as a linear predictor per 10% increment. A consistent protective association was observed across all models, with hazard ratios ranging from 0.39 to 0.72 (**Supplementary Table S1**), supporting the validity of the ≥20% threshold as the primary cut-off.

### Tumor–stroma ratio

The tumor–stroma ratio (TSR) was evaluated at the invasive front using a 10× objective lens, following the method described by Mesker et al. (30). The microscopic field with the highest proportion of stromal area was selected for assessment. Tumors were classified as stroma-high (low TSR) when stromal tissue occupied >50% of the selected field, and as stroma-low (high TSR) when stromal tissue accounted for ≤50%.

### Tumor budding

Tumor budding was defined as the presence of a single tumor cell or a cluster of fewer than five tumor cells at the invasive front. Assessment was conducted following the recommendations of the International Tumor Budding Consensus Conference (ITBCC 2016) (15). Buds were counted in a single hotspot field at 20× magnification, and tumor budding was categorized as low (0–4 buds), intermediate (5–9 buds), or high (≥10 buds).

### Statistical analysis

The primary endpoint was progression-free survival (PFS), defined as the interval from initiation of ICI therapy to documented disease progression or death from any cause. Secondary endpoints included objective response rate (ORR; complete or partial response) and overall survival (OS).

Inter-observer agreement for sTILs, TSR, and tumor budding was evaluated using Cohen’s kappa coefficient. Survival curves were generated using the Kaplan–Meier method and compared with the log-rank test. Multivariable analyses were performed using Cox proportional hazards regression models to identify independent prognostic factors for PFS, with results reported as hazard ratios (HRs) and 95% confidence intervals (CIs).

All statistical analyses were conducted using R software (version 4.2.0), and a two-sided P value <0.05 was considered statistically significant.

## Results

### Baseline clinicopathological characteristics

A total of 210 patients were included in the final analysis. The median age was 59 years (interquartile range [IQR], 48–68), and 61.0% (n=128) were male. Primary tumors were located in the left colon or rectum in 140 patients (66.7%) and in the right colon in 70 patients (33.3%). Regarding molecular subtypes, 48 patients (22.9%) were classified as dMMR, while 162 patients (77.1%) were classified as pMMR.

**Figure 1:**
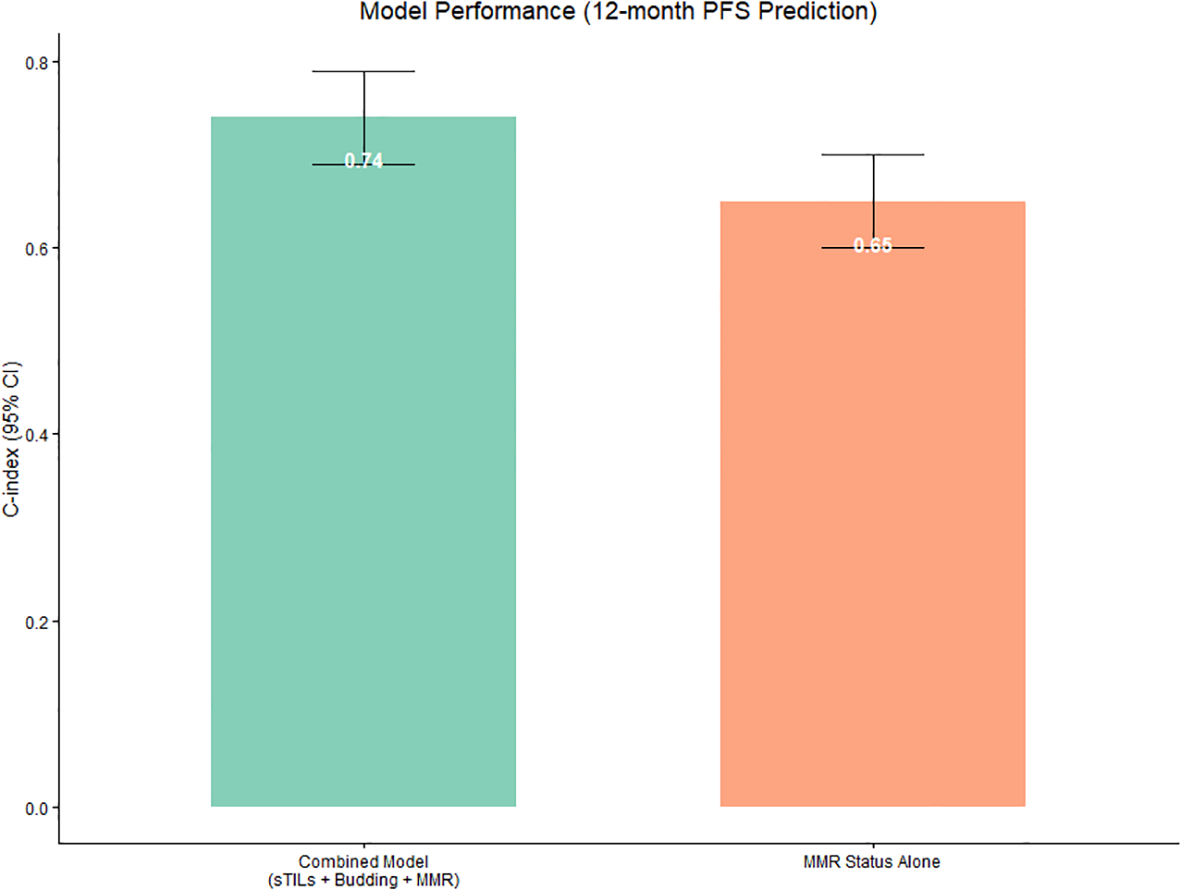
Representative H&E-stained sections illustrating scoring extremes for the three histopathological biomarkers (20× objective). **(A)** High sTILs (≥20%): dense stromal lymphocytic infiltrate. **(B)** Low sTILs (<20%): sparse infiltrate. **(C)** Stroma-low TSR (≥50% tumour area): tumour-predominant invasive front. **(D)** Stroma-high TSR (<50% tumour area): abundant desmoplastic stroma. **(E)** Low-grade tumour budding (Bd1, 0–4 buds per 0.785 mm²). **(F)** High-grade tumour budding (Bd3, ≥10 buds per 0.785 mm²). Scale bars = 100 μm. H&E, haematoxylin and eosin; sTILs, stromal tumour-infiltrating lymphocytes; TSR, tumour–stroma ratio; Bd, budding grade (ITBCC 2016).

Histopathological evaluation showed that 72 patients (34.3%) had high stromal tumor-infiltrating lymphocytes (sTILs ≥20%), and 95 patients (45.2%) exhibited a high stromal component (tumor–stroma ratio [TSR] <50%). High-grade tumor budding (Bd 3) was observed in 55 patients (26.2%). Inter-observer agreement for histopathological assessment was substantial to excellent, with kappa values of 0.86 for sTILs, 0.84 for TSR, and 0.82 for tumor budding (**Table 1**). Representative H&E-stained images illustrating the scoring extremes for each biomarker are shown in **Figure 1**.

**Table 1:** Baseline clinicopathological characteristics of 210 patients with aCRC stratified by sTILs status.

Characteristic	Total (N = 210)	High sTILs (n = 72)	Low sTILs (n = 138)	*P* value
Age, median (IQR), years	59 (48–68)	57 (45–66)	60 (50–71)	0.142
Sex, n (%)				0.725
Male	128 (61.0)	45 (62.5)	83 (60.1)	
Female	82 (39.0)	27 (37.5)	55 (39.9)	
Primary tumor site, n (%)				0.038*
Left-sided colon/rectum	140 (66.7)	41 (56.9)	99 (71.7)	
Right-sided colon	70 (33.3)	31 (43.1)	39 (28.3)	
MMR status, n (%)				<0.001*
dMMR	48 (22.9)	28 (38.9)	20 (14.5)	
pMMR	162 (77.1)	44 (61.1)	118 (85.5)	
Tumor–stroma ratio (TSR), n (%)				0.012*
Low stroma (TSR ≥ 50%)	115 (54.8)	49 (68.1)	66 (47.8)	
High stroma (TSR < 50%)	95 (45.2)	23 (31.9)	72 (52.2)	
Tumor budding, n (%)				0.024*
Bd 1–2 (low/intermediate)	155 (73.8)	61 (84.7)	94 (68.1)	
Bd 3 (high)	55 (26.2)	11 (15.3)	44 (31.9)	

sTILs, stromal tumor-infiltrating lymphocytes; TSR, tumor–stroma ratio; MMR, mismatch repair; dMMR, deficient mismatch repair; pMMR, proficient mismatch repair; IQR, interquartile range.

High sTILs were defined as ≥20%.

Low stromal content was defined as TSR ≥50%.

P values were calculated using the χ² test or Fisher’s exact test for categorical variables and the Mann–Whitney U test for continuous variables.

### Correlation between histopathological features and treatment response

The objective response rate (ORR) for the entire cohort was 26.2% (55/210). Patients with high sTILs demonstrated a significantly higher ORR compared with those with low sTILs (47.2% vs. 15.2%, P<0.001). Similarly, tumors with low stromal content (TSR ≥50%) were associated with a superior ORR compared with high-stroma tumors (36.5% vs. 13.7%, P<0.001).

**Figure 2:**
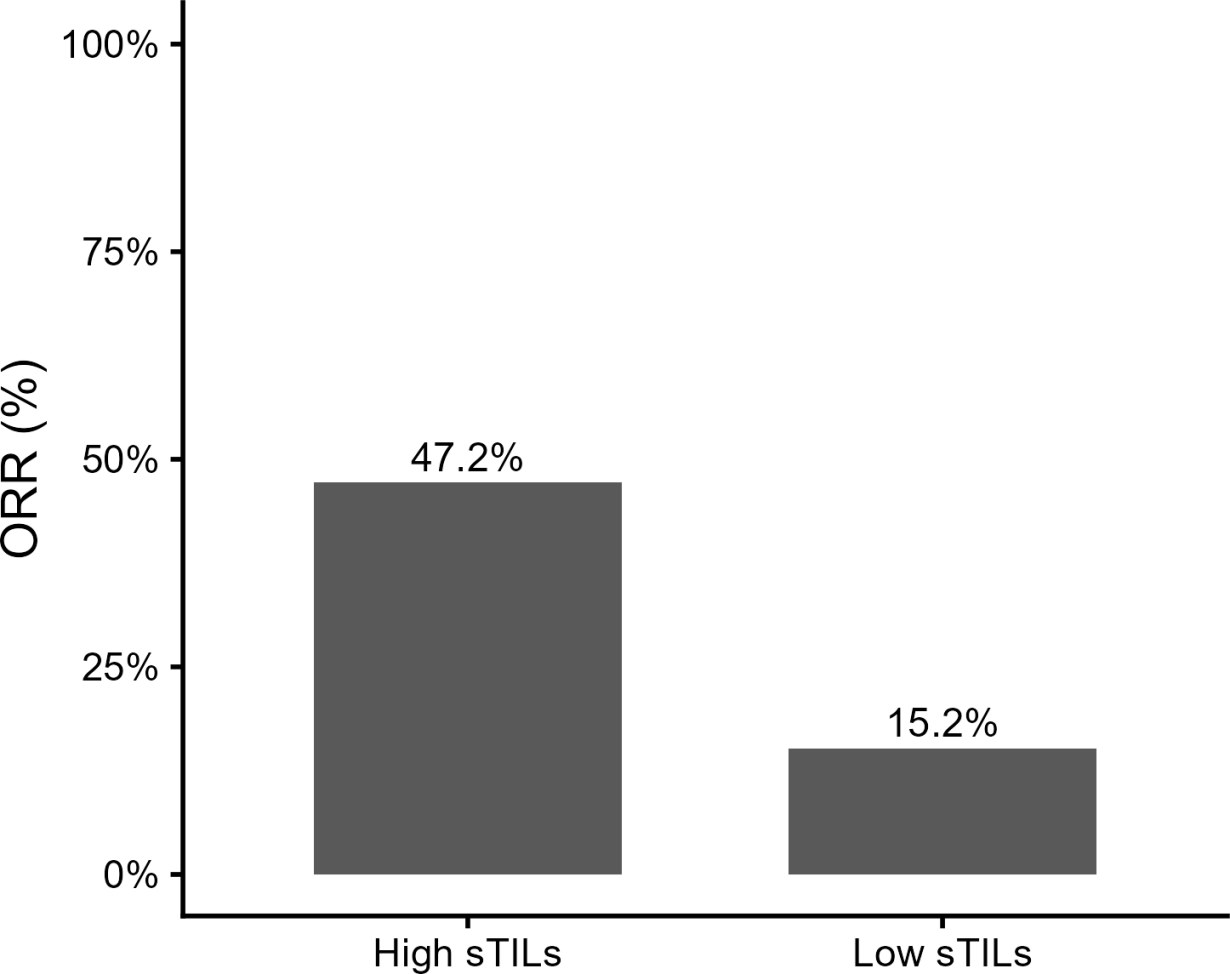
**(A)** ORR by sTILs status in the overall cohort (High sTILs 47.2% vs Low sTILs 15.2%). **(B)** ORR by TSR, stromal content (Low stroma/TSR ≥50%: 36.5% vs High stroma/TSR <50%: 13.7%).

In subgroup analysis restricted to pMMR patients (n=162), the ORR remained significantly higher in the high-sTILs group than in the low-sTILs group (31.4% vs. 8.4%, P<0.001). Notably, when sTILs and TSR were evaluated in combination, patients with both high sTILs and low stromal content—defined as an immune-active phenotype—achieved an ORR of 38.5%, even within the pMMR population (**Figure 2**). By contrast, pMMR patients with neither feature had an ORR of 6.1% (95% CI, 2.3–13.0%), underscoring the clinical relevance of this combined immune-active phenotype (P<0.001 for comparison). A dedicated multivariable Cox proportional hazards analysis restricted to the pMMR subgroup (n=162), adjusting for age, sex, tumor location, and treatment regimen, confirmed high sTILs (HR 0.51, 95% CI 0.26–0.99, P = 0.048) and high-grade tumor budding (HR 2.18, 95% CI 1.42–3.35, P<0.001) as independent prognostic factors for PFS within this subgroup, while TSR did not achieve independent significance (HR 0.79, 95% CI 0.47–1.34, P = 0.383).

### Survival analysis: progression-free survival

The median follow-up duration was 22.4 months. The median progression-free survival (PFS) for the overall cohort was 6.4 months (95% CI, 5.8–7.2).

**Figure 3:**
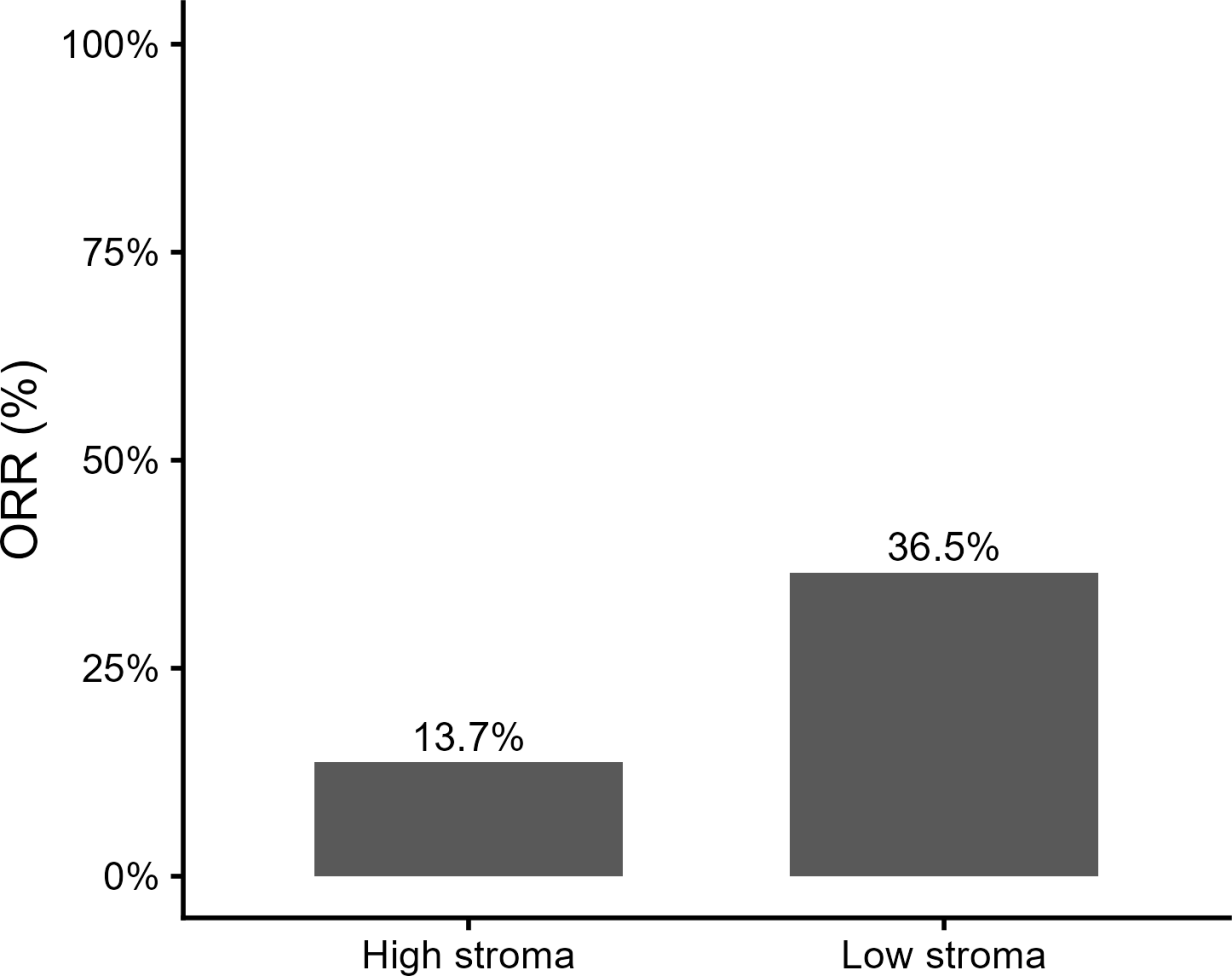
Kaplan–Meier curves for PFS stratified by sTILs, TSR, and tumor budding.

Kaplan–Meier analysis demonstrated that patients with high sTILs experienced significantly longer median PFS compared with those with low sTILs (14.2 months vs. 4.8 months; log-rank P<0.0001). Patients with high stromal content (TSR <50%) had significantly shorter median PFS than those with low stromal content (5.2 months vs. 10.8 months; log-rank P<0.001). In addition, high-grade tumor budding was associated with significantly shorter PFS compared with low or intermediate tumor budding (median 3.9 months vs. 8.1 months, Log-rank P = 0.004) (**Figure 3**).

### Overall survival

Overall survival (OS) was a prespecified secondary endpoint. However, at the current median follow-up of 22.4 months, the number of OS events was insufficient for reliable Kaplan–Meier estimation or multivariable Cox regression (the OS event rate in this cohort had not yet reached the threshold required for stable hazard ratio estimation). Accordingly, mature OS data are not reported in the present analysis. A planned follow-up assessment at 36 months from last patient enrollment will provide sufficient events for definitive OS reporting, and these results will be presented in a subsequent publication.

### Multivariate analysis of prognostic factors

**Figure 4:**
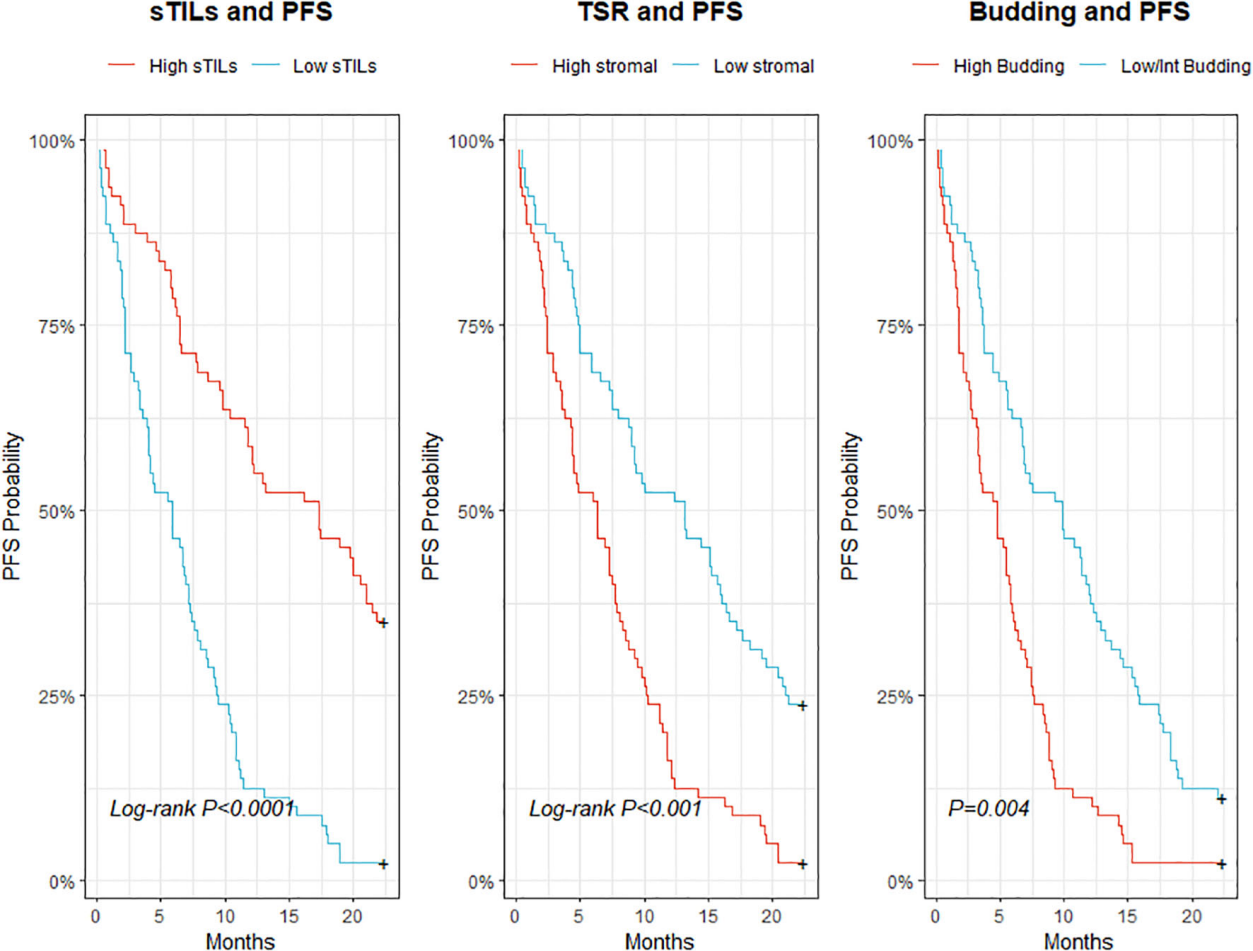
Multivariable Cox regression forest plot for PFS, adjusted for age, sex, tumor location, MMR status, treatment regimen, and line of therapy. High sTILs: HR 0.47 (95% CI 0.26–0.84); Bd3: HR 2.03 (95% CI 1.39–2.96); TSR: not independently significant (HR 0.71, P = 0.154).

To determine whether histopathological features independently predicted ICI efficacy, multivariable Cox proportional hazards regression analyses were performed after adjustment for age, sex, primary tumor location, and MMR status. High sTILs (HR 0.47, 95% CI 0.26–0.84, P = 0.011) and high-grade tumor budding (HR 2.03, 95% CI 1.39–2.96, P<0.001) remained independent prognostic factors for PFS. However, low stromal content (TSR ≥50%) did not maintain independent significance in the multivariable model (HR 0.71, 95% CI 0.45–1.14, P = 0.154), potentially due to its biological correlation with lymphocyte infiltration (**Figure 4**).

**Figure 5:**
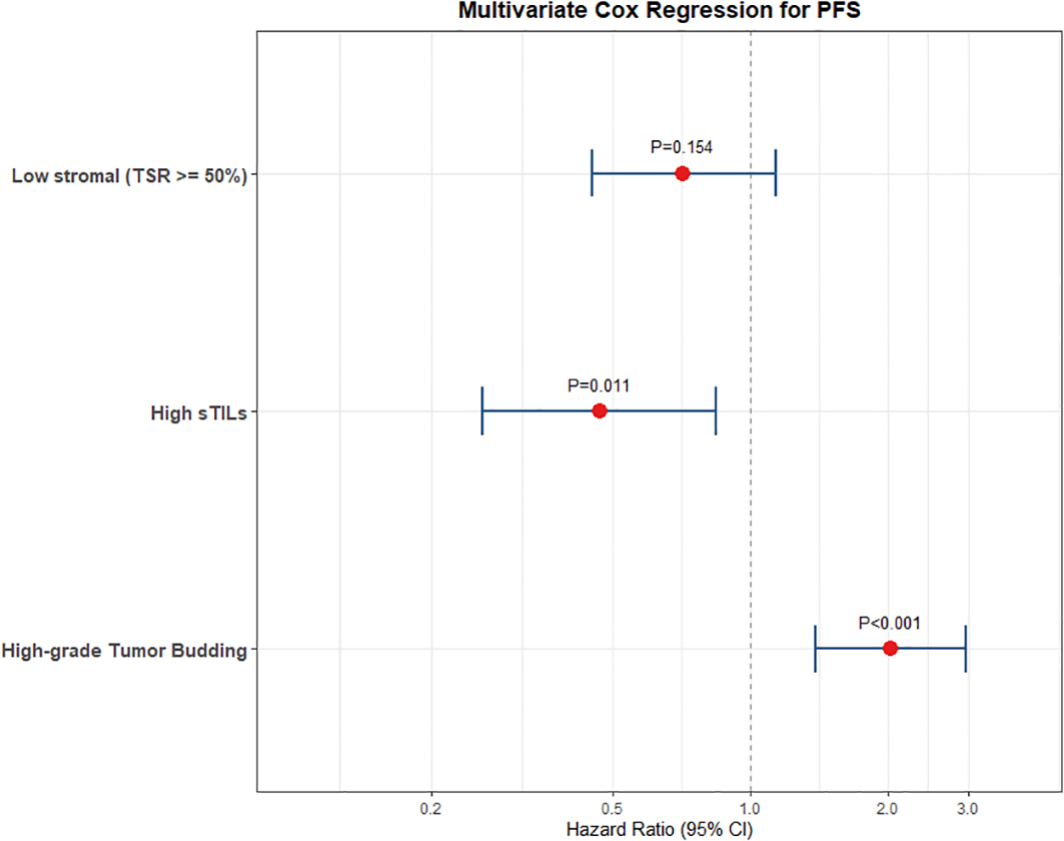
Discriminative performance of the combined model for 12-month PFS (C-index 0.74) compared with MMR status alone (C-index 0.65).

The combined model incorporating sTILs, tumor budding, and MMR status demonstrated superior discriminative performance for predicting 12-month PFS, with a C-index of 0.74 (95% CI, 0.69–0.79), exceeding that of MMR status alone (C-index 0.65) (**Figure 5**).

## Discussion

In this multicentre retrospective analysis of 210 patients with advanced colorectal cancer receiving immune checkpoint inhibitors, we demonstrate that routine histopathological features specifically sTILs, tumor–stroma ratio, and tumor budding are independently associated with immunotherapy efficacy and progression-free survival. Importantly, these associations persisted after adjustment for established clinical variables, including MMR status, highlighting the potential of morphology-based biomarkers to complement molecular stratification strategies in aCRC.

Among the evaluated parameters, stromal tumor-infiltrating lymphocytes emerged as a robust prognostic marker strongly associated with treatment outcome in ICI-treated patients. Patients with high sTILs exhibited markedly higher objective response rates and prolonged PFS, even within the pMMR subgroup. These findings support the concept that immune infiltration is not exclusively determined by genomic instability but may also arise from alternative mechanisms, such as localized antigenicity, microbiome-driven inflammation, or epigenetic modulation (19, 20). Morphologically, high sTILs likely represent a pre-existing, albeit restrained, anti-tumor immune response that can be reinvigorated by PD-1/PD-L1 blockade. This observation aligns with prior reports linking immune-inflamed phenotypes to immunotherapy sensitivity across multiple tumor types (21, 22), while extending their relevance to aCRC patients traditionally considered immunotherapy-refractory.

The tumor–stroma ratio demonstrated complementary prognostic value in univariate analysis, though it did not retain independent significance in the multivariable model. High stromal content (low TSR) was associated with inferior ORR and shorter PFS, consistent with the notion of stromal-mediated immune exclusion. The desmoplastic stroma not only constitutes a physical barrier that impedes lymphocyte trafficking but also functions as an active immunosuppressive compartment through cancer-associated fibroblast signaling, extracellular matrix remodeling, and cytokine secretion, particularly via the TGF-β pathway (23–25). Our results suggest that patients with stroma-rich tumors may derive limited benefit from ICI monotherapy, supporting emerging therapeutic strategies that combine immune checkpoint blockade with stroma-targeting or TGF-β–modulating agents (26).

In contrast to some previous reports, TSR was not an independent predictor in our multivariate model. This discrepancy might be explained by the strong association between a sparse stroma and high sTILs in our cohort. A ‘stroma-rich’ environment may act as a physical barrier that limits T-cell recruitment, suggesting that the prognostic impact of TSR is partially mediated through the immune microenvironment rather than being a purely independent driver of ICI efficacy.

Tumor budding, a histopathological hallmark of epithelial–mesenchymal transition, further refined risk stratification in our cohort. High-grade budding independently predicted poorer PFS, reinforcing its role as a marker of aggressive tumor biology and immune escape. EMT-associated tumor cells exhibit reduced antigen presentation and increased resistance to immune-mediated cytotoxicity, thereby undermining the effectiveness of T-cell–based therapies (27). The coexistence of low sTILs, high stromal content, and extensive tumor budding may therefore define a particularly immunotherapy-resistant phenotype within aCRC.

Several previous studies have established the prognostic relevance of tumor-infiltrating lymphocytes in colorectal cancer. The NCCTG N0147/Alliance analyses by Lee, Sinicrope, and colleagues evaluated resected stage III colon cancer treated in the adjuvant setting and showed that TIL density, particularly when combined with tumor budding, could refine survival prediction (28). The later analysis by Saberzadeh-Ardestani et al. further demonstrated that the association between TIL density and survival may depend on primary tumor sidedness in stage III colon cancer (29). Similarly, Ohtani reviewed the biological and prognostic importance of TILs in colorectal cancer (30), Fuchs et al. validated the International TILs Working Group scoring system as a prognostic tool in a large primary CRC resection cohort (31), Nazemalhosseini-Mojarad et al. linked intratumoral lymphocytes with improved survival independent of oncogenetic features (32), Vitorino et al. assessed TILs in resected stage II and III CRC, and Wang et al. evaluated TIL density in colorectal cancer liver metastases (33). In contrast, the present study specifically focuses on patients with advanced, unresectable or metastatic colorectal cancer receiving PD-1/PD-L1-based immunotherapy. Our endpoints were immunotherapy-related outcomes, including objective response rate and progression-free survival, rather than postoperative disease-free or overall survival alone. In addition, we evaluated a combined H&E-based histopathological framework incorporating sTILs, TSR, and tumor budding, and we specifically examined whether these features could stratify outcomes within the clinically challenging pMMR subgroup. Therefore, our study should be interpreted as extending prior prognostic observations into the real-world immunotherapy-treated aCRC setting, rather than duplicating earlier studies in surgically treated localized CRC (34).

A notable strength of our study lies in the accessibility and reproducibility of the evaluated biomarkers. Unlike advanced molecular assays, sTILs, TSR, and tumor budding can be assessed on standard H&E-stained sections, with excellent inter-observer agreement demonstrated in our analysis. This positions histopathological assessment as a cost-effective and scalable approach for immunotherapy stratification, particularly in resource-limited settings. Nevertheless, several limitations warrant consideration. First, the retrospective design and treatment heterogeneity including variation in ICI agents (Sintilimab, Tislelizumab, and Camrelizumab), partner therapies (chemotherapy, anti-angiogenic agents, and dual ICI combinations), and line of treatment (first- through third-line and beyond) are important limitations that may introduce residual confounding. These factors could independently influence ORR and PFS, and their effects cannot be fully disentangled in the current analysis despite sensitivity adjustments. Second, as all patients received ICI-based treatment and no non-ICI comparator arm was included, the associations observed between histopathological features and outcomes reflect prognostic stratification within treated patients rather than true predictive value in the classical sense; a non-ICI control arm would be required to establish genuine predictive utility. Third, OS data were immature at the time of analysis (median follow-up 22.4 months; insufficient events for reliable estimation) and are not reported herein; mature OS data will be presented upon completion of planned follow-up at 36 months from last enrollment. Fourth, the present study does not include benchmarking against transcriptomic or genomic predictive tools for ICI response, such as tumour mutational burden, consensus molecular subtype (CMS) classification, or gene expression profiling. These molecular approaches represent increasingly validated biomarker strategies in colorectal cancer immunotherapy, and their absence from the current analysis reflects the limitations of routine clinical practice at the participating centres during the enrolment period; future prospective studies integrating histopathological scoring with comprehensive molecular profiling will be required to determine the incremental and complementary predictive value of the markers described here relative to established genomic classifiers. Finally, prospective validation in independent cohorts is required to confirm the generalizability of these findings. Integration with digital pathology and artificial intelligence–based quantification may further enhance the objectivity and clinical utility of these markers in future studies.

## Conclusion

In conclusion, standardized evaluation of routine histopathological features provides meaningful prognostic and outcome-stratifying information for immunotherapy outcomes in advanced colorectal cancer. High sTILs, low stromal content, and low tumor budding independently identify patients more likely to benefit from immune checkpoint inhibitors within treated cohorts, including subsets within pMMR disease. These readily available, cost-effective biomarkers offer a practical complement to molecular testing and may facilitate more refined, pathology-driven patient selection for personalized immunotherapy strategies, pending prospective validation with comparative trial designs.

## Data Availability

The raw data supporting the conclusions of this article will be made available by the authors, without undue reservation.

## References

[1] Sung H Ferlay J Siegel RL . Global cancer statistics 2020: GLOBOCAN estimates of incidence and mortality worldwide for 36 cancers in 185 countries. CA Cancer J Clin. (2021) 71:209–49. doi: 10.3322/caac.21660 33538338

[2] Arnold M Sierra MS Laversanne M Soerjomataram I Jemal A Bray F . Global patterns and trends in colorectal cancer incidence and mortality. Gut. (2017) 66:683–91. doi: 10.1136/gutjnl-2015-310912 26818619

[3] Le DT Durham JN Smith KN . Mismatch repair deficiency predicts response of solid tumors to PD-1 blockade. Science. (2017) 357:409–13. doi: 10.1126/science.aan6733 28596308 PMC5576142

[4] Overman MJ McDermott R Leach JL . Nivolumab in patients with metastatic DNA mismatch repair–deficient or microsatellite instability–high colorectal cancer (CheckMate 142). Lancet Oncol. (2017) 18:1182–91. doi: 10.1016/S1470-2045(17)30422-9 28734759 PMC6207072

[5] André T Shiu KK Kim TW . Pembrolizumab in microsatellite-instability–high advanced colorectal cancer. N Engl J Med. (2020) 383:2207–18. doi: 10.1056/NEJMoa2017699 33264544

[6] Le DT Uram JN Wang H . PD-1 blockade in tumors with mismatch-repair deficiency. N Engl J Med. (2015) 372:2509–20. doi: 10.1056/NEJMoa1500596 26028255 PMC4481136

[7] Cohen R Hain E Buhard O . Association of primary resistance to immune checkpoint inhibitors in metastatic colorectal cancer with misdiagnosis of microsatellite instability or mismatch repair deficiency status. JAMA Oncol. (2019) 5:551–5. doi: 10.1001/jamaoncol.2018.4942 30452494 PMC6459114

[8] Chan TA Yarchoan M Jaffee E . Development of tumor mutation burden as an immunotherapy biomarker: utility for the oncology clinic. Ann Oncol. (2019) 30:44–56. doi: 10.1093/annonc/mdy495 30395155 PMC6336005

[9] Litchfield K Reading JL Puttick C . Meta-analysis of tumor- and T cell–intrinsic mechanisms of sensitization to checkpoint inhibition. Cell. (2021) 184:596–614.e14. doi: 10.1016/j.cell.2021.01.002 33508232 PMC7933824

[10] Pagès F Mlecnik B Marliot F . International validation of the consensus Immunoscore for the classification of colon cancer: a prognostic and accuracy study. Lancet. (2018) 391:2128–39. doi: 10.1016/S0140-6736(18)30789-X 29754777

[11] Binnewies M Roberts EW Kersten K . Understanding the tumor immune microenvironment (TIME) for effective therapy. Nat Med. (2018) 24:541–50. doi: 10.1038/s41591-018-0014-x 29686425 PMC5998822

[12] Fridman WH Zitvogel L Sautès-Fridman C Kroemer G . The immune contexture in cancer prognosis and treatment. Nat Rev Clin Oncol. (2017) 14:717–34. doi: 10.1038/nrclinonc.2017.101 28741618

[13] Hendry S Salgado R Gevaert T . Assessing tumor-infiltrating lymphocytes in solid tumors: a practical review for pathologists and proposal for a standardized method from the International Immuno-Oncology Biomarkers Working Group: Part 2. Adv Anat Pathol. (2017) 24:311–35. doi: 10.1097/PAP.0000000000000161 28777143 PMC5638696

[14] Park JH Richards CH McMillan DC Horgan PG Roxburgh CSD . The relationship between tumour stroma percentage, the tumour microenvironment and survival in patients with primary operable colorectal cancer. Ann Oncol. (2014) 25:644–51. doi: 10.1093/annonc/mdt593 24458470 PMC4433525

[15] Lugli A Kirsch R Ajioka Y . Recommendations for reporting tumor budding in colorectal cancer based on the International Tumor Budding Consensus Conference (ITBCC) 2016. Mod Pathol. (2017) 30:1299–311. doi: 10.1038/modpathol.2017.46 28548122

[16] Graham RP Vierkant RA Tillmans LS . Tumor budding in colorectal carcinoma: confirmation of prognostic significance and histologic cutoff in a population-based cohort. Am J Surg Pathol. (2015) 39:1340–6. doi: 10.1097/PAS.0000000000000504 26200097 PMC4567920

[17] Salgado R Denkert C Campbell C . Tumor-infiltrating lymphocytes and associations with pathological complete response and event-free survival in HER2-positive early-stage breast cancer treated with lapatinib and trastuzumab. JAMA Oncol. (2015) 1:448–54. doi: 10.1001/jamaoncol.2015.0830 26181252 PMC5551492

[18] Mlecnik B Van den Eynde M Bindea G . Comprehensive intrametastatic immune quantification and major impact of immunoscore on survival. J Natl Cancer Inst. (2018) 110:97–108. doi: 10.1093/jnci/djx123 28922789

[19] Gopalakrishnan V Helmink BA Spencer CN Reuben A Wargo JA . The influence of the gut microbiome on cancer, immunity, and cancer immunotherapy. Cancer Cell. (2018) 33:570–80. doi: 10.1016/j.ccell.2018.03.015 29634945 PMC6529202

[20] Routy B Le Chatelier E Derosa L . Gut microbiome influences efficacy of PD-1–based immunotherapy against epithelial tumors. Science. (2018) 359:91–7. doi: 10.1126/science.aan3706 29097494

[21] Cristescu R Mogg R Ayers M . Pan-tumor genomic biomarkers for PD-1 checkpoint blockade–based immunotherapy. Science. (2018) 362:eaar3593. doi: 10.1126/science.aar3593 30309915 PMC6718162

[22] Ayers M Lunceford J Nebozhyn M . IFN-γ–related mRNA profile predicts clinical response to PD-1 blockade. J Clin Invest. (2017) 127:2930–40. doi: 10.1172/JCI91190 28650338 PMC5531419

[23] Mariathasan S Turley SJ Nickles D . TGFβ attenuates tumour response to PD-L1 blockade by contributing to exclusion of T cells. Nature. (2018) 554:544–8. doi: 10.1038/nature25501 29443960 PMC6028240

[24] Kalluri R . The biology and function of fibroblasts in cancer. Nat Rev Cancer. (2016) 16:582–98. doi: 10.1038/nrc.2016.73 27550820

[25] Chen X Song E . Turning foes to friends: targeting cancer-associated fibroblasts. Nat Rev Drug Discov. (2019) 18:99–115. doi: 10.1038/s41573-018-0004-1 30470818

[26] Lan Y Zhang D Xu C . Enhanced preclinical antitumor activity of M7824, a bifunctional fusion protein simultaneously targeting PD-L1 and TGF-β. Sci Transl Med. (2018) 10:eaan5488. doi: 10.1126/scitranslmed.aan5488 29343622

[27] Dongre A Weinberg RA . New insights into the mechanisms of epithelial–mesenchymal transition and implications for cancer. Nat Rev Mol Cell Biol. (2019) 20:69–84. doi: 10.1038/s41580-018-0080-4 30459476

[28] Lee H Sha D Foster NR . Analysis of tumor microenvironmental features to refine prognosis by T, N risk group in patients with stage III colon cancer (NCCTG N0147) (Alliance). Ann Oncol. (2020) 31:487–98. doi: 10.1016/j.annonc.2020.01.011 32165096 PMC7372727

[29] Saberzadeh-Ardestani B Foster NR Lee HE Shi Q Alberts SR Smyrk TC . Association of tumor-infiltrating lymphocytes with survival depends on primary tumor sidedness in stage III colon cancers (NCCTG N0147) [Alliance. Ann Oncol. (2022) 33:1159–67. doi: 10.1016/j.annonc.2022.07.1942 35963480 PMC9882989

[30] Ohtani H . Focus on TILs: prognostic significance of tumor infiltrating lymphocytes in human colorectal cancer. Cancer Immun. (2007) 7:4. 17311363 PMC2935759

[31] Fuchs TL Sioson L Sheen A . Assessment of tumor-infiltrating lymphocytes using International TILs Working Group system is a strong predictor of overall survival in colorectal carcinoma: a study of 1034 patients. Am J Surg Pathol. (2020) 44:536–44. doi: 10.1097/PAS.0000000000001409 31743129

[32] Nazemalhosseini-Mojarad E Mohammadpour S Torshizi Esafahani A . Intratumoral infiltrating lymphocytes correlate with improved survival in colorectal cancer patients: independent of oncogenetic features. J Cell Physiol. (2019) 234:4768–77. doi: 10.1002/jcp.27273 30370522

[33] Vitorino M Eiriz I Tomás TC . Association of tumor-infiltrating lymphocytes with survival in stages II and III colorectal cancer. Cureus. (2022) 14:e31144. doi: 10.7759/cureus.31144 36505147 PMC9728984

[34] Wang E Shibutani M Nagahara H . Prognostic value of the density of tumor-infiltrating lymphocytes in colorectal cancer liver metastases. Oncol Lett. (2021) 22:837. doi: 10.3892/ol.2021.13098 34712361 PMC8548800

