# Supplementary material for: Histopathological biomarkers of immunotherapy outcome in advanced colorectal cancer: a multicentre retrospective study
Source: Front Oncol. 2026 Jul 6;16:1811658. doi: 10.3389/fonc.2026.1811658 (PMC13381254; doi:10.3389/fonc.2026.1811658)
Supplement: Supplementary file 1 [file Table1.docx]

**Supplementary Table S1**

*Sensitivity analyses of the sTILs threshold for progression-free survival (PFS): multivariable Cox proportional hazards models using alternative cut-offs and a continuous-variable model (overall cohort, n=210).*

| **sTILs Threshold** | **n High (%)** | **HR (95% CI)†** | **P value** |
| --- | --- | --- | --- |
| ≥10% (low threshold) | ~116 (55.2) | 0.58 (0.30–1.12) | 0.104 |
| ≥20% (primary, pre-specified) | 72 (34.3) | 0.47 (0.26–0.84) | 0.011 |
| ≥30% (high threshold) | ~45 (21.4) | 0.39 (0.18–0.83) | 0.014 |
| Continuous (per 10% increment) | — | 0.72 (0.64–0.81) | <0.001 |

**Footnotes**

† All Cox models adjusted for age, sex, primary tumor location, MMR status, treatment regimen, and line of therapy.

‡ The ≥10% cut-off produces a confidence interval crossing the null (HR 0.58, 95% CI 0.30–1.12, P=0.104), reflecting dilution of the effect by borderline cases. The continuous-variable model (HR 0.72 per 10% increment, P<0.001) confirms a consistent dose-response association across the full sTILs range.

Abbreviations: sTILs, stromal tumor-infiltrating lymphocytes; HR, hazard ratio; CI, confidence interval; MMR, mismatch repair.
